# Mobilized colistin-resistant gene (MCR-1) in extended-spectrum beta-lactamase-producing clinical isolates of Escherichia coli in Abuja, Nigeria

**DOI:** 10.3205/dgkh000671

**Published:** 2026-09-01

**Authors:** Muhammad Abdullahi, Aisha Shehu Adamu, Lawan Adamu, Rebecca Olajumoke Bolaji, Jimoh Olanrewaju, Busayo Olalekan Olayinka

**Affiliations:** 1 Department of Pharmaceutical Microbiology, Faculty of Pharmaceutical Sciences, Ahmadu Bello University, Zaria, Nigeria; 2 Department of Microbiology, Federal Medical Centre, Jalingo, Taraba State, Nigeria; 3 Department of Pharmacy, Federal Medical Centre, Jalingo, Taraba State, Nigeria; 4 Department of Planning, Research and Statistics, Nigeria Centre for Disease Control and Prevention (NCDC), HQ Office, Abuja, Nigeria; 5 Health Research Division: Golden Bricks Inc., Nigeria; 6 The Management, Federal Medical Centre, Jalingo, Taraba State, Nigeria; 7 Department of Medical Microbiology, Ahmadu Bello University Teaching Hospital, Zaria, Nigeria

**Keywords:** multiple drug resistance, extended-spectrum beta-lactamase (ESBL), mobilized colistin resistance gene (MCR-1 gene), antimicrobial resistance, antimicrobial stewardship, Nigeria

## Abstract

**Introduction:**

The global increase in reports of transferable colistin resistance has become a significant public health concern. In Nigeria, only a limited number of studies have documented the presence of mobilized colistin resistance genes (MCR) in isolates derived from both human and animal sources.

**Aim:**

This investigation aimed to detect the presence of the mobilized colistin-resistant gene (MCR-1) in multiple drug-resistant, extended-spectrum beta-lactamase-producing clinical isolates of *Escherichia (E.) coli* and *Klebsiella (K.) pneumoniae* obtained from selected hospitals in Abuja, Nigeria.

**Materials and method:**

A total of 115 consecutive, non-duplicate presumptive clinical isolates of *E. coli* and *K. pneumoniae* were collected over three months from two hospitals in Abuja: Garki Hospital Abuja and Nisa Hospital Abuja. These isolates were identified employing rapid identification kits. Antimicrobial susceptibility testing was conducted using the agar disc diffusion method, while phenotypic detection of ESBLs was performed via double-disc synergy tests. The MIC of colistin was determined using the broth microdilution method. Molecular characterization of the ESBL and MCR-1 genes was achieved through PCR analysis.

**Results:**

Out of 115 clinical isolates, 49 (42.6%) were identified as *E. coli* and *K. pneumoniae*, with 36 (73.5%) of these being multidrug-resistant (MDR). Among the MDR isolates, 15 demonstrated phenotypic resistance to third-generation cephalosporins (primary indicators for TEM and SHV-derived ESBLs), e.g., cefpodoxime. These 15 isolates were selected for ESBL screening. Seven isolates tested positive and were subsequently subjected to double-disc synergy testing. Notably, five out of these seven isolates were confirmed as phenotypic ESBL producers. The result of PCR analysis revealed that 3/5 harbored multiple ESBL genes; *E. coli* harbored blaSHV and *bla*CTX-M or *bla*SHV and *bla*TEM, *K. penumoniae* harbored *bla*SHV, *bla*TEM, and *bla*CTX-M. None of the investigated isolates harbored the *bla*OXA gene. A total of 8 isolates comprising the 3 genotypic ESBL-positive isolates, the 2 phenotypic colistin-resistant isolates, and 3 borderline phenotypic colistin-resistant isolates were molecularly analyzed to detect MCR-1 genes. One out of /8 isolates of *E. coli* isolated from a patient’s urine sample harbored the MCR-1 gene.

**Conclusion:**

This study is, to the best of our knowledge, the first report of MCR-1 gene detection from a human clinical sample in Abuja, the capital city of Nigeria. The increasing emergence of mobilized colistin resistance genes in isolates of clinical significance as such, calls for an urgent need to preserve the efficacy of our last resort antibiotics through the institutionalization and adequate implementation of antibiotic stewardship.

## Zusammenfassung

**Einleitung:**

Der weltweite Anstieg von Berichten über übertragbare Colistinresistenz unterstreicht ein erhebliches Problem für die öffentliche Gesundheit. In Nigeria wurde das Vorkommen mobilisierter Colistinresistenzgene (MCR) in Isolaten humanen und tierischen Ursprungs bisher nur in wenigen Studien dokumentiert.

**Ziel:**

Diese Untersuchung hatte zum Ziel, das Vorkommen des mobilisierten Colistinresistenzgens (MCR-1) in multiresistenten, ESBL-bildenden klinischen Isolaten von *Escherichia (E.) coli* und *Klebsiella (K.) pneumoniae* aus ausgewählten Krankenhäusern in Abuja, Nigeria, nachzuweisen.

**Methode:**

Über einen Zeitraum von drei Monaten wurden 115 aufeinanderfolgende, nicht duplizierte, mutmaßliche klinische Isolate von *E. coli* und *K. pneumoniae* aus zwei Krankenhäusern in Abuja gesammelt. Die Identifizierung der Isolate erfolgte mittels Schnelltests. Die antibiotische Empfindlichkeit wurde mittels Agardiffusionstest bestimmt, während der phänotypische Nachweis von ESBL über Doppelscheiben-Synergietests erfolgte. Die minimale Hemmkonzentration (MHK) von Colistin wurde mittels Mikrodilutionsverfahrens ermittelt. Die molekulare Charakterisierung der ESBL- und MCR-1-Gene erfolgte durch PCR-Analyse.

**Ergebnisse:**

Von 115 klinischen Isolaten wurden 49 (42,6%) als *E. coli* und *K. pneumoniae* identifiziert, davon waren 36 (73,5%) multiresistent (MDR). Unter den MDR-Isolaten zeigten 15 eine phänotypische Resistenz gegen Cephalosporine der dritten Generation wie Ceftazidim und Cefotaxim sowie gegen Cefpodoxim. Diese 15 Isolate wurden für das ESBL-Screening ausgewählt. Sieben Isolate fielen positiv aus und wurden anschließend einem Doppelscheiben-Synergietest unterzogen. Fünf dieser sieben Isolate wurden als phänotypische ESBL-Produzenten bestätigt. Die PCR-Analyse ergab, dass 3/5 mehrere ESBL-Gene trugen; *E. coli* trug die Gene *bla*SHV und *bla*CTX-M bzw. *bla*SHV und *bla*TEM, *K. pneumoniae* die Gene *bla*SHV, *bla*TEM und *bla*CTX-M. Keines der untersuchten Isolate trug das Gen *bla*OXA. Insgesamt wurden acht Isolate – drei genotypisch ESBL-positive, zwei phänotypisch Colistin-resistente und drei phänotypisch grenzwertig Colistin-resistente Isolate – molekularbiologisch auf das Vorhandensein des MCR-1-Gens untersucht. Eins der acht Isolate aus der Urinprobe eines Patienten trug das MCR-1-Gen.

**Schlussfolgerung:**

Die Studie ist unseres Wissens der erste Bericht über den Nachweis des MCR-1-Gens in einer humanen klinischen Probe in Abuja, der Hauptstadt Nigerias. Angesichts des zunehmenden Auftretens mobilisierter Colistin-Resistenzgene in klinisch relevanten Isolaten ist es dringend erforderlich, die Wirksamkeit der Reserveantibiotika durch Institutionalisierung und adäquate Umsetz ung eines rationalen Antibiotikaeinsatzes zu erhalten.

## Introduction

In the past decade, the World Health Organization has included colistin (Polymyxin E) on its list of essential medicines [1]. Now, in 2026, colistin remains a critical last-resort antibiotic for the treatment of life-threatening infections caused by multidrug-resistant (MDR) Gram-negative bacteria [2]. The global escalation of carbapenem-resistant Enterobacteriaceae (CRE) and extended-spectrum beta-lactamase (ESBL)-producing pathogens, combined with a stagnant pipeline for novel antibiotics, has led to a resurgence in colistin use [3]. However, its clinical utility is increasingly challenged by the rapid global dissemination of both chromosomal and plasmid-mediated resistance [4].

Today, the challenge is further complicated by the rapid spread of plasmid-mediated resistance genes (MCR-1 to MCR-10), which facilitate horizontal transmission across clinical, environmental, and agricultural sectors (one health) at a global scale [5]. In Nigeria, in the past decade, only a handful of studies have described colistin resistance across the one-health spectrum. This research study aimed at investigating the presence of the mobilized colistin-resistant gene (MCR-1) in multiple-drug-resistant, ESBL-producing clinical isolates of *E. coli *and* Klebsiella pneumoniae* from some hospitals in Abuja, Nigeria.

A significant component of surveillance studies for the control of antimicrobial resistance is, of course, ongoing surveillance to characterize circulating resistance phenotypes and detect new resistance genes and their spread in important human pathogens. This will help in policy making to combat antimicrobial resistance.

## Materials and methods

### Settings and study design

The clinical isolates analyzed in this descriptive epidemiological study were collected between June and August 2018 at the Garki Hospital, Abuja (GHA), and Nisa Hospital, Abuja (NHA) in Nigeria. The Federal Capital Territory Health Research Ethics Committee (FCT HREC) reviewed and granted ethical clearance for this research (FHREC/2018/01/22/02-03-18).

### Sample collection and identification

115 non-duplicate clinical isolates of *E. coli *and* K. pneumonia* obtained from urine and stool samples of patients attending the two hospitals were collected for this study. These isolates were cultured and preliminarily identified using the standard microbiological techniques (colony morphology, Gram stain, oxidase, and various biochemical tests). The identity of the isolates was confirmed using the rapid identification kit Microgen GN-ID (product code: MID-64CE, Microgen GN-ID A, UK). 

### ESBL

The susceptibility of the identified isolates to commonly prescribed antibiotics was determined using the modified Kirby-Bauer disc diffusion method as described in the 2018 EUCAST guideline [6]. The following antibiotics were used in this study: ampicillin (10 µg), amoxi-clav (20:10 µg), cefotaxime (30 µg), cefpodoxime (10 µg), ceftazidime (30 µg), colistin (150 mg), imipenem (10 µg), aztreonam (30 µg), gentamicin (10 µg), ofloxacin (5 µg) and nitrofurantoin (300 µg). The minimum inhibitory concentration (MIC) of colistin was determined by the micro-broth dilution method, as previously described [6]. The isolates presumptively identified as ESBL producers by their resistance to ceftazidime, cefotaxime, and cefpodoxime were subjected to a confirmatory double-disc synergy test as described by the European Committee on Antimicrobial Susceptibility Testing [7]. Briefly, the dual-disc synergy test (DDST) was performed by placing amoxycillin/clavulanic acid (AMC) discs at the center of a Mueller–Hinton agar plate inoculated with a 0.5 McFarland standard turbidity suspension of the test organism. Then, ceftazidime (30 µg) and cefotaxime (30 µg) discs were placed around the AMC disc (20 mm apart, center-to-center). The plate was then inverted and incubated at 37°C for 16–18 hours. The DDST was considered positive when the inhibition zone of either antibiotic expanded toward the centrally placed AMC.

### Molecular detection of ESBL and MRC genes

The phenotypically ESBL-positive isolates and isolates with reduced susceptibility to colistin were screened for the common ESBL (*bla*SHV, *bla*TEM, *bla*CTX-M, and *bla*OXA) and mobilized colistin resistance (MCR-1) genes using the conventional polymerase chain reaction technique.

### DNA extraction

The genomic DNA was extracted from an overnight culture of the test isolates using the Zymo Research Bacterial DNA Miniprep Kit (Zymo Research, Irvine, CA) following the manufacturer’s instructions. The obtained DNA was then subjected to 1% agarose gel electrophoresis to ascertain its quality.

### PCR technique

The PCR reactions was carried out in a GeneAmp1 PCR System 9700 thermocycler (Thermo Fisher Scientific) set to a pre-optimized condition [8]. After the PCR cycles, 5 mL of the resulting PCR products were subjected to 2% agarose gel electrophoresis, stained with 10 mg/ml ethidium bromide. and were then visualized by ultraviolet trans- illumination. A 1-kb DNA Ladder (Thermo Fisher Scientific) was used as a molecular weight marker during electrophoresis. The primers used in this study are listed in Table 1 (Tab. 1). 

### Data analysis

The data collected were analyzed using IBM SPSS software, version 26. Descriptive analysis, including frequencies and percentages, was used. 

## Results

### Isolation rates of the clinical isolates

The distribution of the 115 presumptive clinical isolates comprising 77 presumptive *Escherichia (E.) coli* and 38 *K. pneumoniae* was obtained from patients attending the two hospitals (Table 2 (Tab. 2)).

### Biochemical identification of the clinical isolates

The percentage distribution of the 49/115 (42.6%) identified *E. coli *and* K. pneumoniae* clinical isolates obtained from the two hospitals (Table 3 (Tab. 3)).

### Antimicrobial susceptibility testing

36/49 of the clinical isolates subjected to antibiotic susceptibility testing were multidrug-resistant isolates and were therefore categorized according to Magiorakos et al. [9] as multiple drug-resistant (MDR, n=18), extremely drug-resistant (XDR, n=6), and pan-drug-resistant (PDR, n=2) (Figure 1 (Fig. 1)).

### Antibiotic resistance profile of the MDR clinical isolates

The *E. coli* strains obtained from the Garki Hospital Abuja were mainly resistant to ceftazidime, cefpodoxime, ofloxacin, nitrofurantoin, and cefotaxime. In the Nisa Hospital Abuja the proportion of resistant *E. coli* strains were higher (Table 4 (Tab. 4)). For *K. pneumoniae*, there was no notable difference in resistance between the isolates from the two hospitals (Table 4 (Tab. 4)). 

MIC results for the clinical isolates: Two of 49 colistin-resistant isolates had MIC values above the 4.0 µg/ml threshold. Three of 49 other isolates had MIC values at exactly 2.0 µg/ml and hence were termed “borderline colistin-resistant isolates” (Table 5 (Tab. 5)).

### Phenotypic detection of ESBL in MDR clinical isolates

Fifteen of 36 MDR isolates of E. coli and K. pneumoniae obtained from GHA and NHA – comprising 12 isolates from GHA and 3 from NHA that have shown phenotypic resistance to the third-generation cephalosporins such as ceftazidime and cefotaxime (the best indicator for TEM and SHV-derived ESBL) and cefpodoxime (the best indicator for all ESBL types) – were selected for ESBL screening. Seven of 15 of clinical isolates tested for ESBL preliminary disc screening were positive. However, when further subjected to the phenotypic confirmatory DDST, Five of 7 were phenotypically confirmed ESBL producers (Table 6 (Tab. 6)).

The five identified MDR phenotypically extended ESBL-producing clinical isolates of *E. coli* and *K. pneumoniae* obtained in the two hospitals are shown in Table 7 (Tab. 7).

### Molecular characterization of these ESBL genes (SHV, TEM, CTX-M, and OXA)

The five identified MDR phenotypically ESBL-producing clinical isolates were further subjected to a conventional PCR for the molecular characterization of some of the most common clinically significant ESBL genes (TEM, SHV, OXA, and CTX-M). The molecular detection of these ESBL genes (SHV, TEM, CTX-M, and OXA) via multiplex PCR reveals that 3 of 5 isolates were positive for the SHV genes, 2 of 5 harbored the TEM genes, and 2 of 5 were positive for CTX-M genes with at 295 bp, 404 bp and 754 bp, respectively. The OXA gene was not detected in any of the isolates (Figure 1 (Fig. 1) and Table 8 (Tab. 8)). 

### Molecular characterization of the mobilizing colistin resistance gene (MCR-1 gene)

Isolates harboring the ESBL genes (G5, G15, and G20), colistin-resistant isolates (N03 and G35), and all the borderline colistin-resistant isolates (G8, N11, and G30) were analyzed.

Resolution of the amplification products by agarose gel electrophoresis revealed that the plasmid-encoded colistin resistance gene (MCR-1) evaluated in this study was present in only 1/8 (12.5%), G35, out of a total number of 8 isolates analyzed (Figure 2 (Fig. 2) and Table 9 (Tab. 9)).

## Discussion

The detection of resistant genes is a global public health issue. Surveillance studies such as this, aimed at identifying specific resistant genes, are key in combating antimicrobial resistance. It is well documented that β-lactam antibiotics are the most-prescribed antibiotics in Nigeria [10]. The majority of the isolates exhibited high resistance to β-lactam antibiotics. This may be due to therapeutic dependence on β-lactam antibiotics owing to their high tolerability and efficacy [11].

Moreover, easy over-the-counter access to this important class of antibiotics and the attendant tendency toward misuse make the emergence of β-lactam-resistant Gram-negative bacteria inevitable. The imipenem resistance observed in this study may be due to the expression of metallo-beta-lactamase enzymes [12]. Previous reports have established the occurrence of metallo-beta-lactamase-producing isolates in various regions of Nigeria, even among patients who have not previously received carbapenem treatment [12], [13].

As witnessed in this study, the detection of ESBL genes among MDR clinical isolates of human origin is worrisome. The incidence of infections caused by third-generation cephalosporin-resistant organisms, particularly members of the Enterobacteriaceae family producing various ESBL enzymes, has increased in recent years [14], [15]. This study's finding is consistent with previous studies in Abuja, where clinical and non-clinical bacterial isolates similarly harbor ESBL genes [16], [17].

The detection of an *E. coli* isolate bearing the MCR-1 gene in this study is cause for concern, because the continued spread of the plasmid-borne MCR-1 gene will compromise the clinical usefulness of polymyxins [18]. With no new antibiotics in the pipeline, colistin has been recently classified by the World Health Organization as a critically important antibiotic for human medicine [19]. This is due to the position of colistin as the last-resort antibiotic for treating infections caused by MDR Gram-negative bacteria. The gradual emergence of colistin resistance among Gram-negative bacteria portends an imminent threat of therapeutic failure in human and veterinary health care, and thus substantial additional morbidity and mortality [20]. Previous studies have reported both plasmid and chromosomal resistance mechanisms of colistin in Nigeria [21], [22], [23], [24]. Similarly, MCR genes have been detected in both clinical and non-clinical bacterial isolates in several African countries, including Algeria [25], Tunisia [26], South Africa [27], and Egypt [28]. To the best of our knowledge, this is the first report of plasmid-mediated colistin resistance in Abuja, the capital city of Nigeria.

The finding of this study is alarming because of the localization of both ESBL and MCR genes on conjugative plasmids, which may harbor other resistant determinant genes. In Tunisia, for example, an isolate of *E. coli* co-harboring MCR-1 and *bla*CTX-M-1 genes alongside other antibiotic-resistant genes on an IncHI2 plasmid has been reported [29]. This makes intra- and inter-species transfer highly likely, with the possibility of a nosocomial outbreak of infections due to these MDR bacterial pathogens. Efforts should therefore be made to strengthen infection control practices in hospitals.

## Conclusion

Coordinated antimicrobial stewardship across one health is paramount in the fight against antimicrobial resistance. The findings concerning the increasing rate of MDR-ESBL genes among isolates of clinical significance, such as *E. coli* and *K. pneumoniae*, is cause for global health concern. This study also reported the first detection of the *MCR-1* gene in an MDR clinical isolate of *E. coli* in Abuja, Nigeria.

## Notes

### Author’s ORCID:

- Abdullahi M: https://orcid.org/0009-0003-5414-7841

### Ethical approval 

The study protocol was reviewed and approved by the Federal Capital Territory Health Research Ethics Committee, FCTA-Abuja, Nigeria [Ethical Approval No: FHREC/2018/01/22/02-03-18].

### Funding

None. 

### Acknowledgments

The authors appreciate the management and the entire staff of the Faculty of Pharmaceutical Sciences, Ahmadu Bello University and Teaching Hospital, Zaria, Kaduna State, Nigeria; the management, pharmacy and microbiology departments of Federal Medical Centre, Jalingo, Taraba State, Nigeria; and the management and microbiology department of Garki Hospital, Abuja and Nisa Hospital, Abuja, Nigeria, for their support.

### Competing interests

The authors declare that they have no competing interests.

## Figures and Tables

**Table 1:**
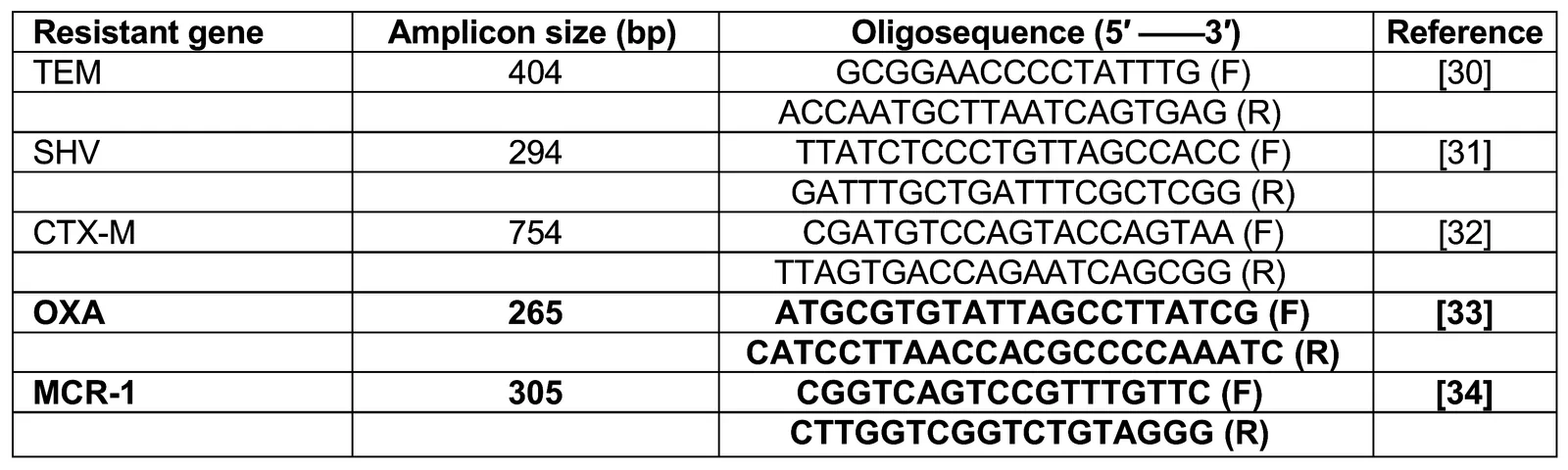
Names and sequences of the primers

**Table 2:**
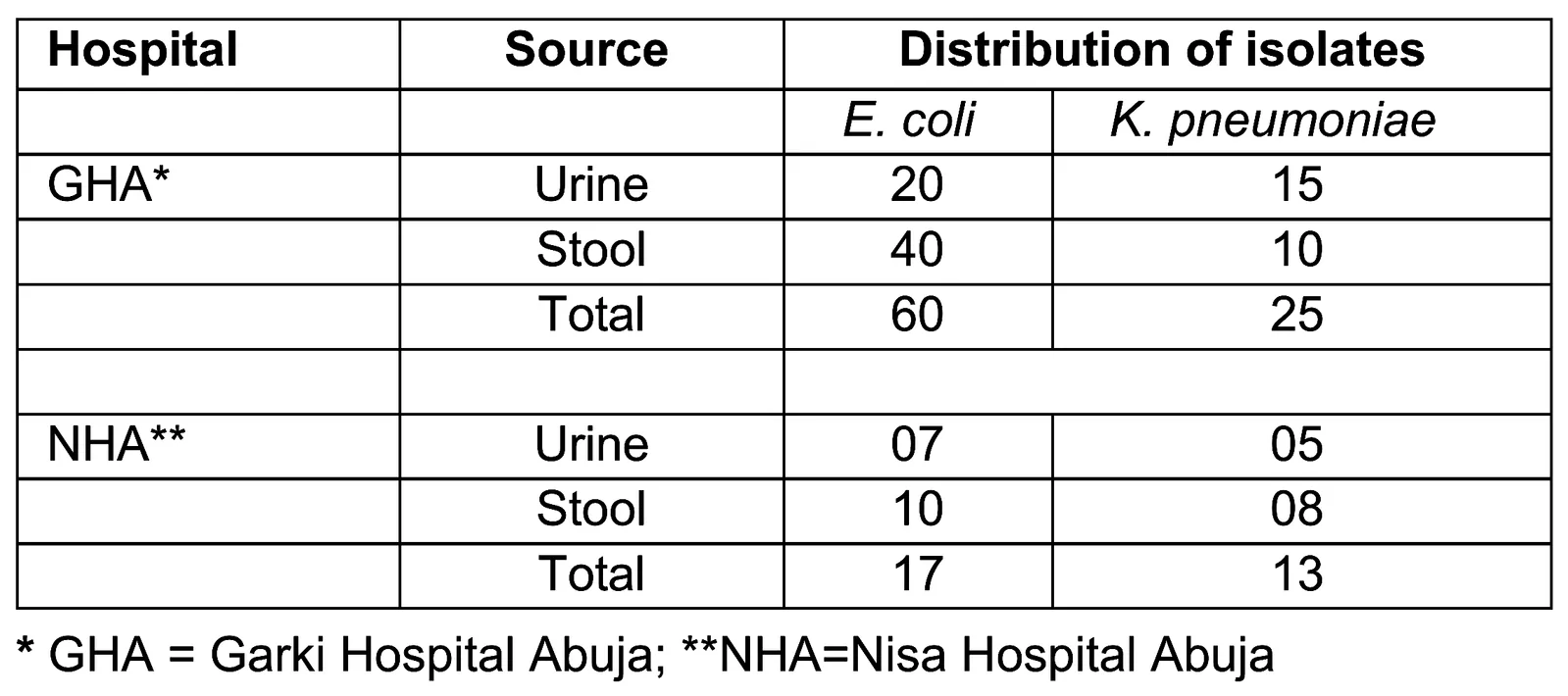
Distribution of the 77 presumptive *E. coli* and 38 *K. pneumoniae* isolates

**Table 3:**
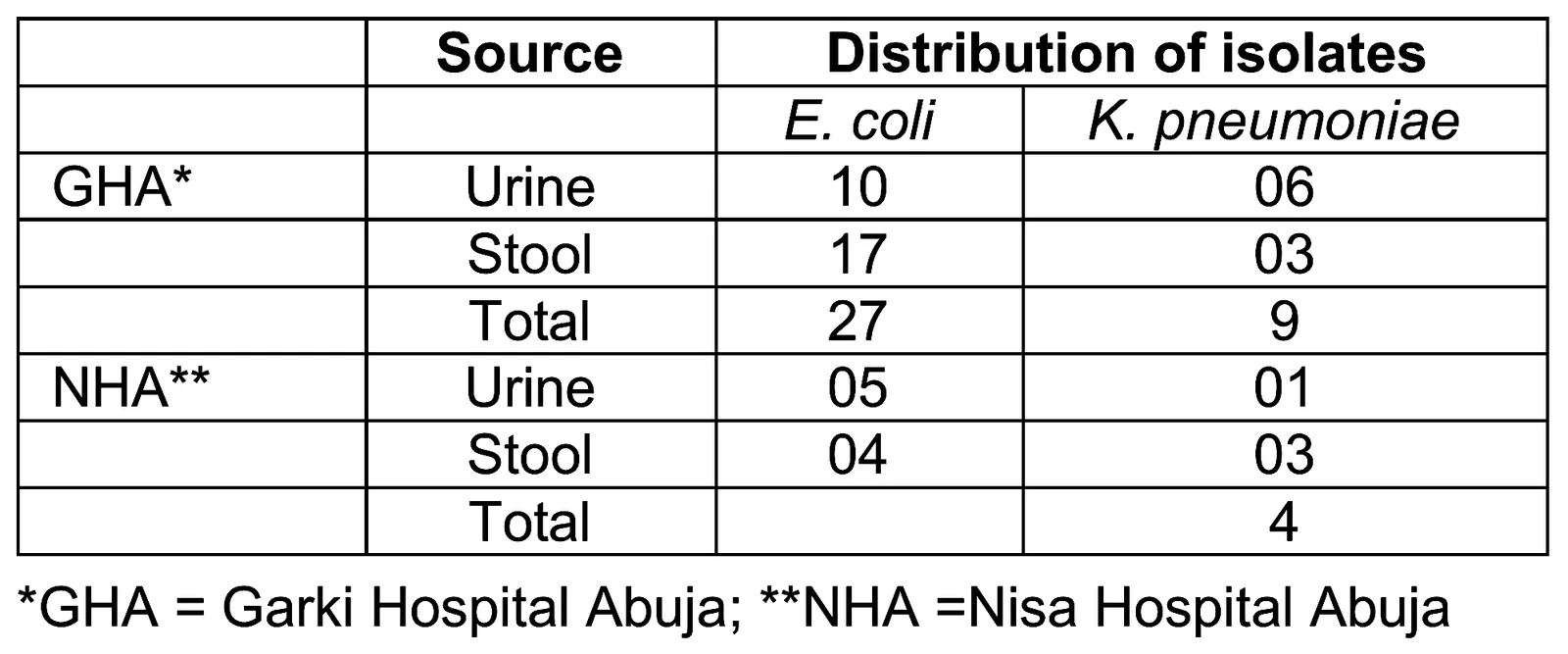
Distribution of identified *E. coli* and *K. pneumoniae* isolates

**Table 4:**
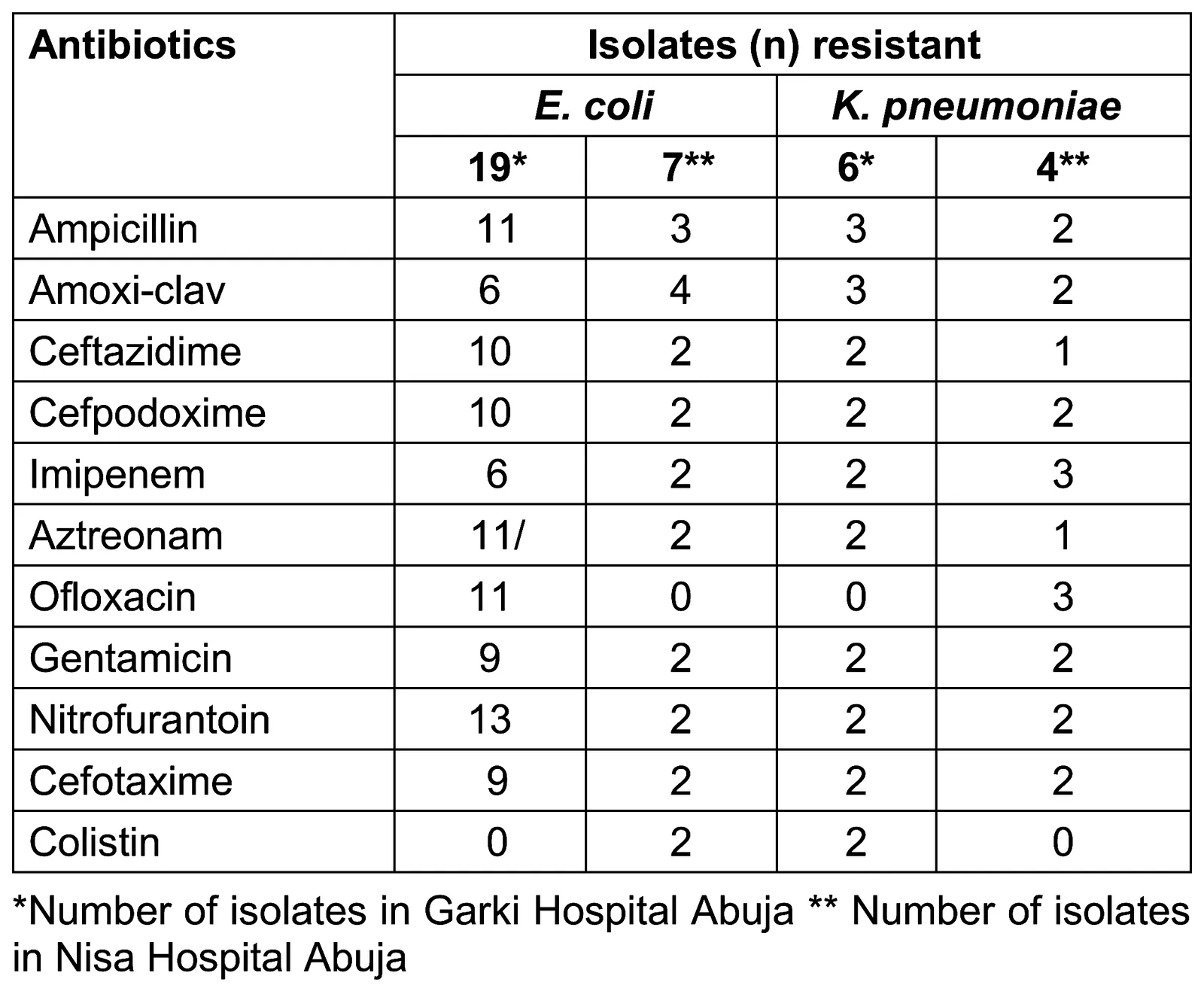
Antibiotic resistance profile of the MDR clinical isolates

**Table 5:**
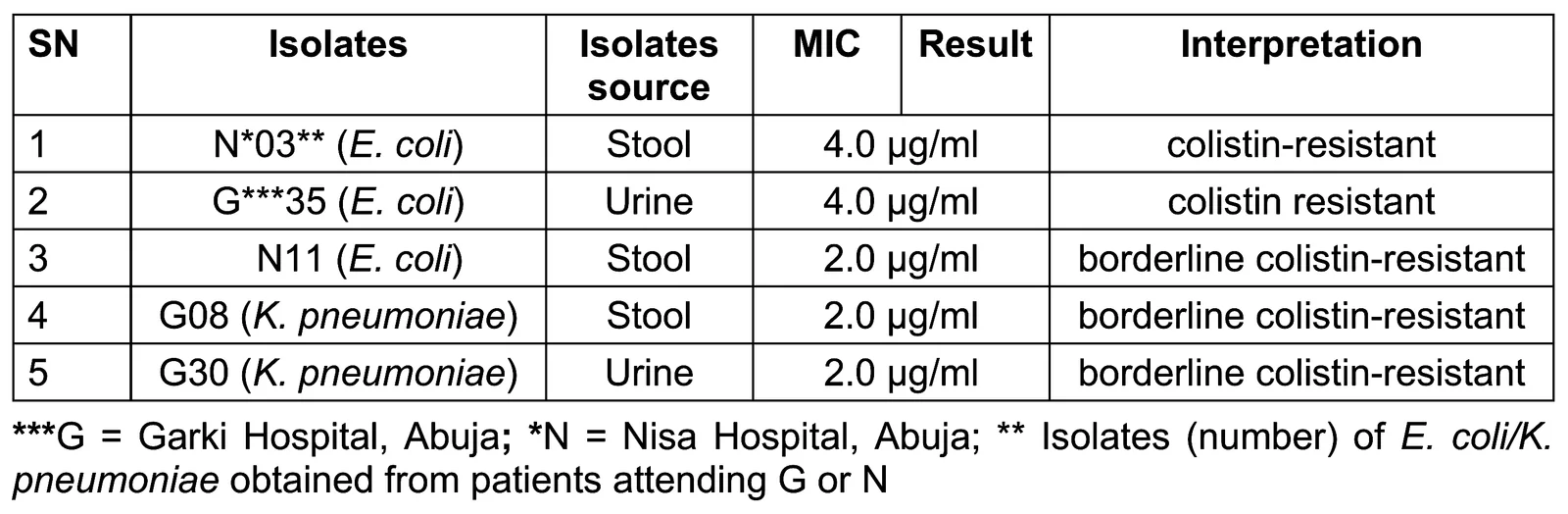
MIC results of the clinical isolates

**Table 6:**
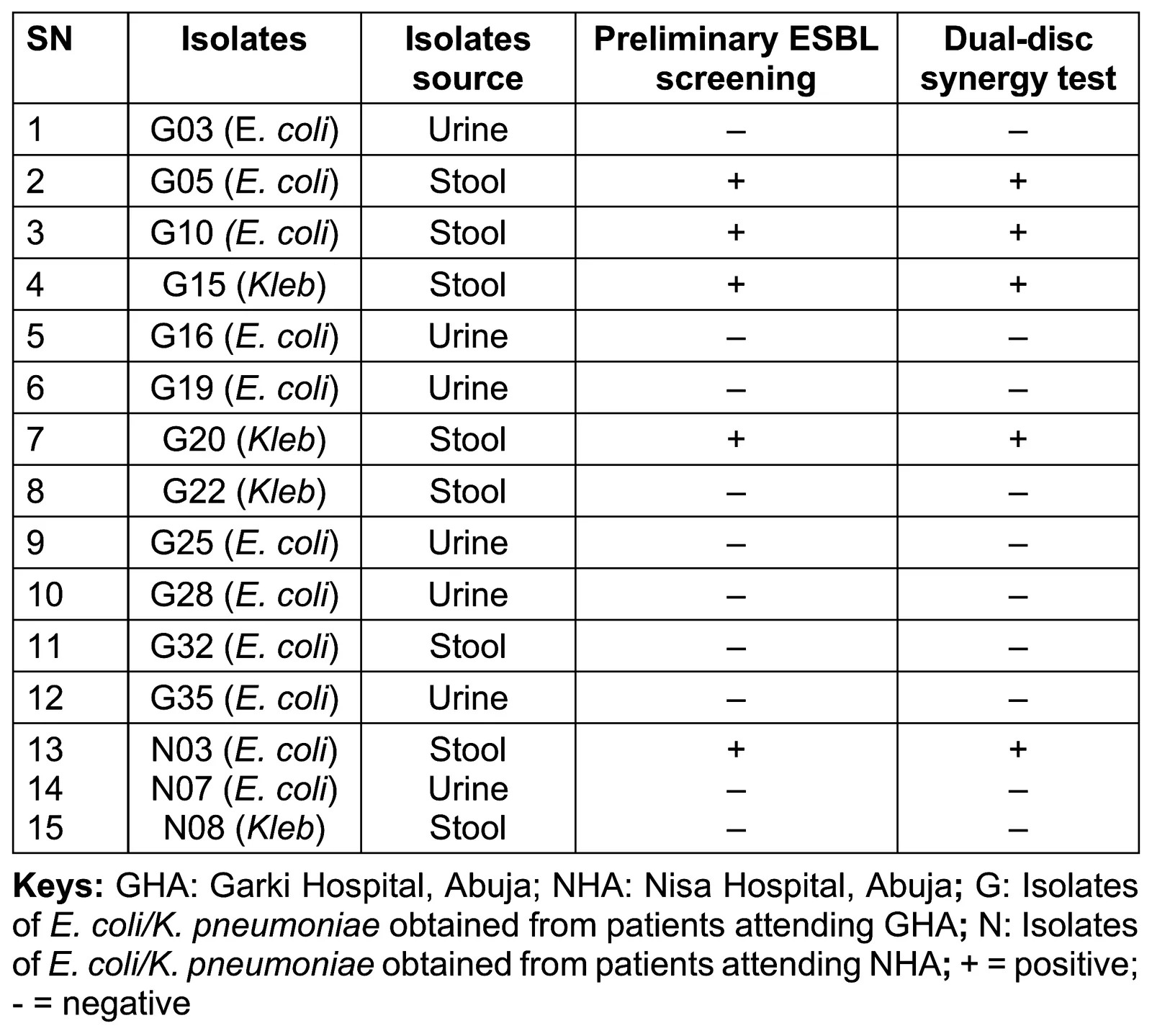
Distribution of phenotypic MDR, ESBL-producing clinical isolates

**Table 7:**
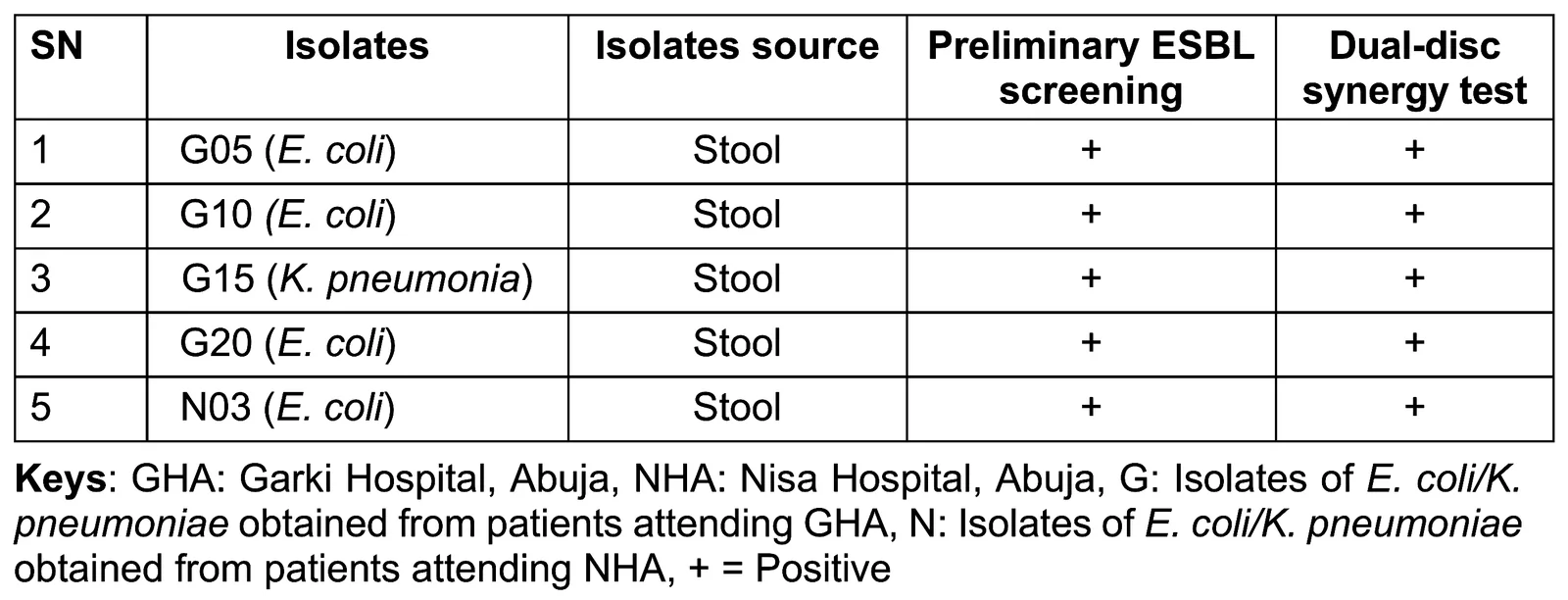
The five identified MDR, phenotypically ESBL -producing clinical isolates

**Table 8:**
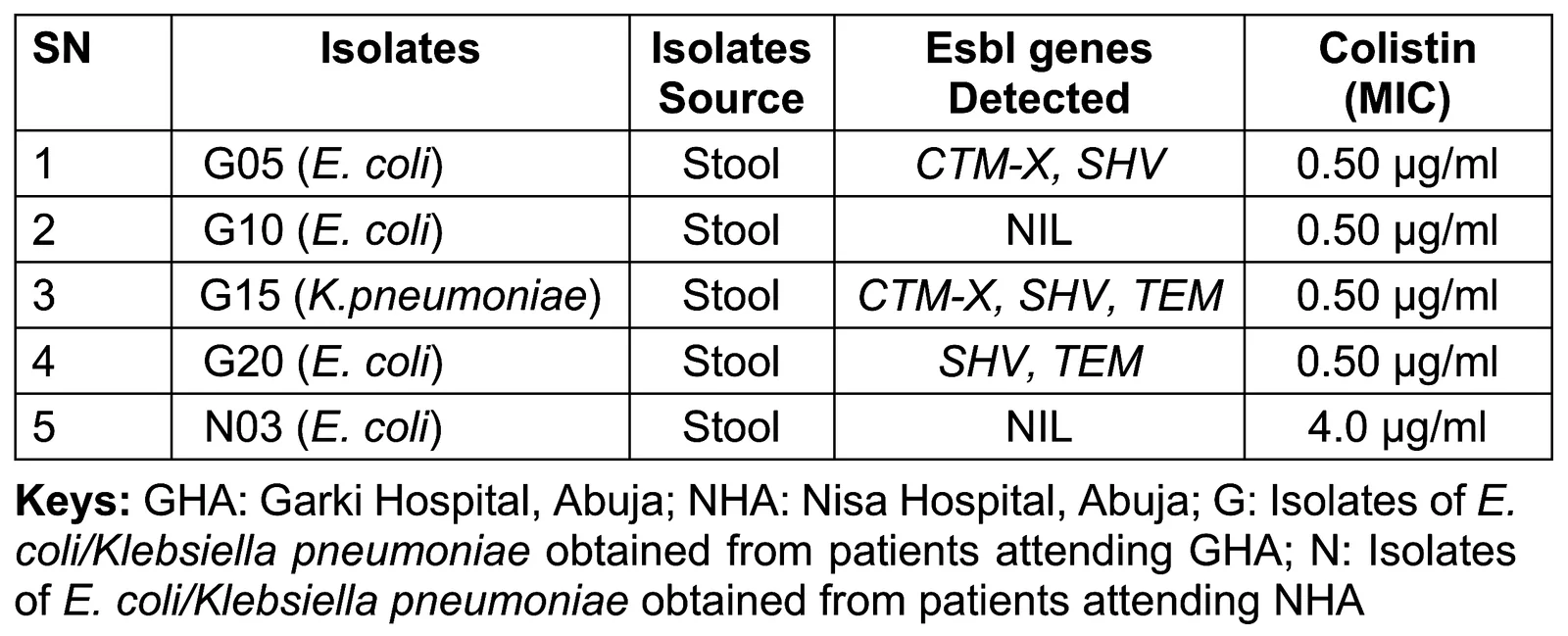
Molecular detection of ESBL genes in MDR *E. coli* and *K. pneumoniae* isolates

**Table 9:**
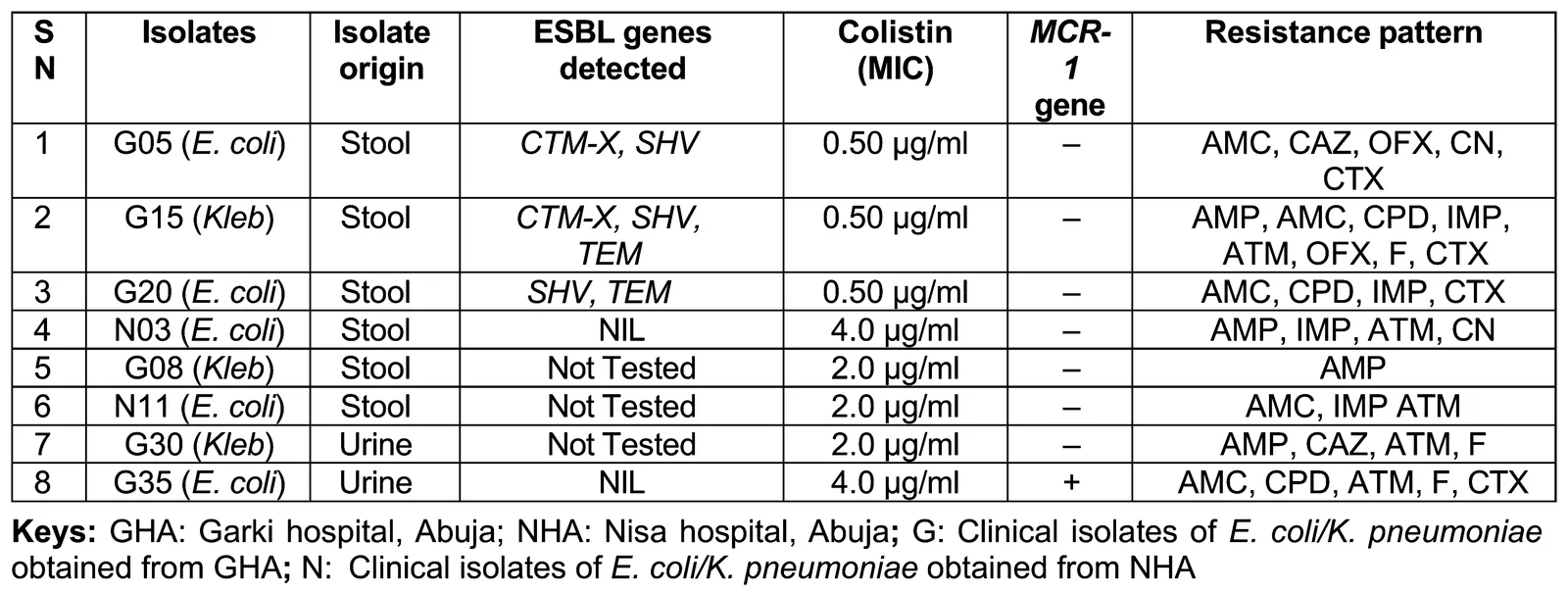
Detection of MCRr-1 genes in MDR ESBL-producing clinical isolates

**Figure 1:**
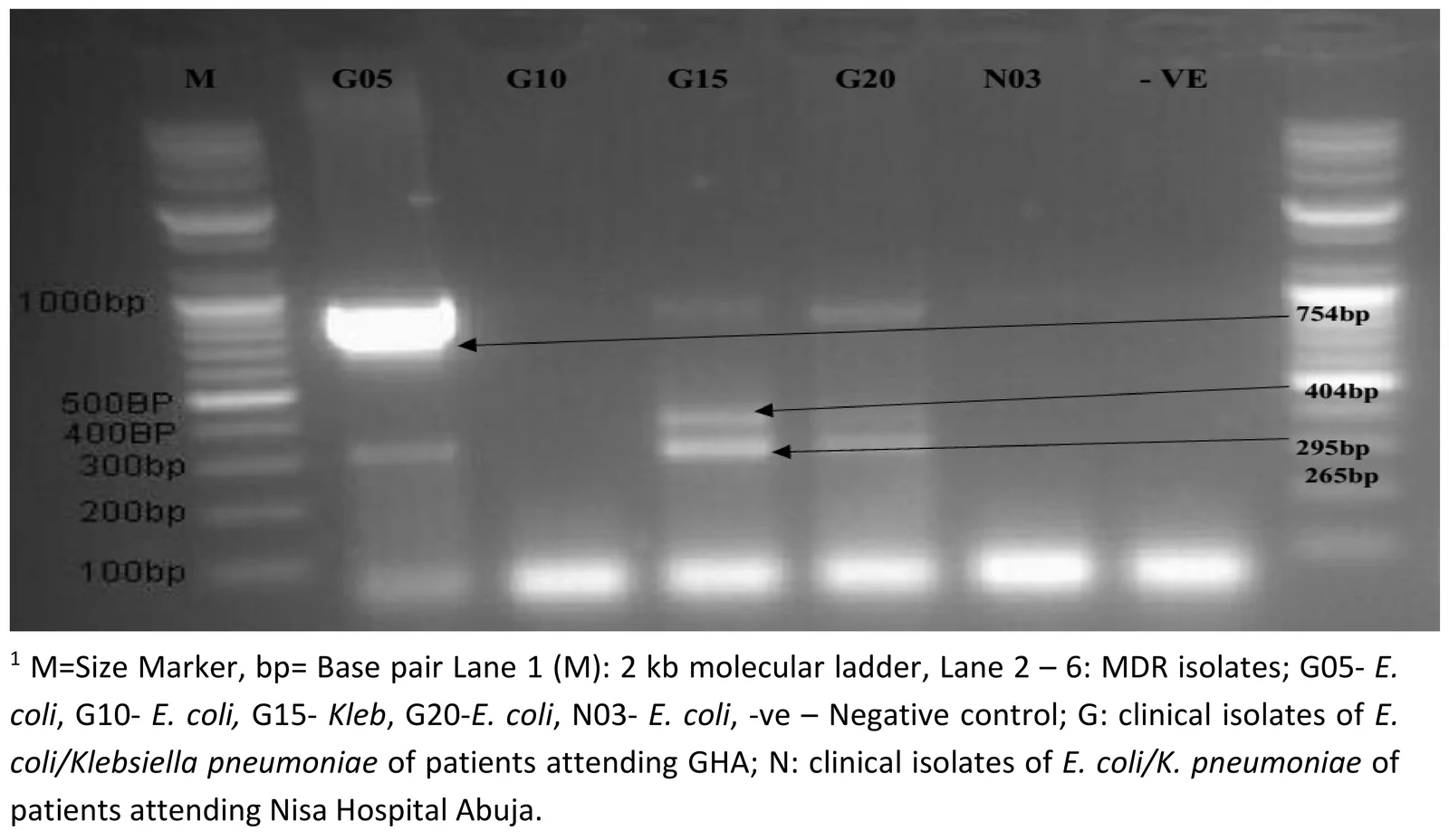
Electrophoretogram of CTX-M (754bp), TEM (404bp), SHV (295bp), and OXA (265bp) genes amplified from MDR *E. coli* and *K. pneumoniae* isolates.

**Figure 2:**
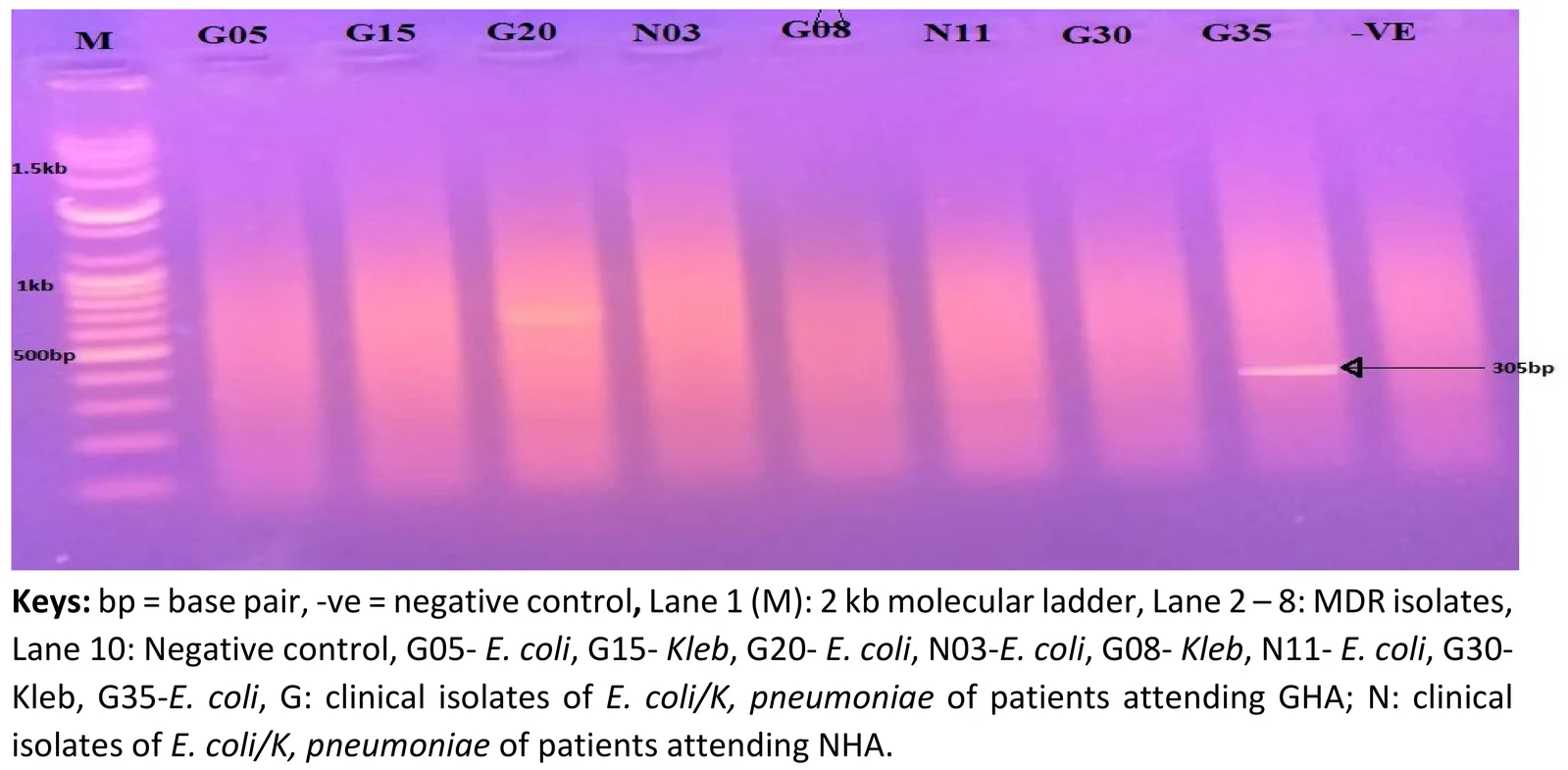
Electrophoretic gel of *MCR-1* (305 bp) gene amplified from MDR, ESBL-producing clinical isolates
